# Modes of administration of nitric oxide devices and ventilators flow-by impact the delivery of pre-determined concentrations

**DOI:** 10.1186/s13613-024-01351-w

**Published:** 2024-08-21

**Authors:** Alice Vuillermoz, Mathilde Lefranc, Nathan Prouvez, Clément Brault, Yoann Zerbib, Mary Schmitt, Jean-Marie Forel, Mathieu Le Tutour, Arnaud Lesimple, Alain Mercat, Jean-Christophe Richard, François M. Beloncle

**Affiliations:** 1 Vent’Lab, Medical Intensive Care Unit, University Hospital of Angers, Angers, France; 2 University of Angers, Angers, France; 3 Med2Lab, Air Liquide Medical Systems, Antony, France; 4 iNOSystems, Antony, France; 5 Medical Intensive Care Unit, Amiens University Hospital, Amiens, France; 6 Air Liquide Santé International, Bagneux, France; 7 Medical Intensive Care Unit, APHM Hôpital Nord, Marseille, France

**Keywords:** Acute respiratory distress syndrome, Artificial lung, Bias flow, byflow, ICU ventilators, Inhaled nitric oxide, iNO delivery systems, Mechanical ventilation, Ventilator flow-by

## Abstract

**Background:**

Nitric oxide (NO) is a strong vasodilator, selectively directed on pulmonary circulation through inhaled administration. In adult intensive care units (ICU), it is mainly used for refractory hypoxemia in mechanically ventilated patients. Several medical delivery devices have been developed to deliver inhaled nitric oxide (iNO). The main purpose of those devices is to guarantee an accurate inspiratory NO concentration, whatever the ventilator used, with NO_2_ concentrations lower than 0.3 ppm. We hypothesized that the performances of the different available iNO delivery systems could depend on their working principle and could be influenced by the ventilator settings. The objective of this study was to assess the accuracy of seven different iNO-devices combined with different ICU ventilators’ flow-by to reach inspiratory NO concentration targets and to evaluate their potential risk of toxicity.

**Methods:**

We tested seven iNO-devices on a test-lung connected to distinct ICU ventilators offering four different levels of flow-by. We measured the flow in the inspiratory limb of the patient circuit and the airway pressure. The nitric oxide/nitrogen (NO/N_2_) flow was measured on the administration line of the iNO-devices. NO and NO_2_ concentrations were measured in the test-lung using an electrochemical analyzer.

**Results:**

We identified three iNO-device generations based on the way they deliver NO flow: “*Continuous*”, “Sequential to inspiratory phase” (*I-Sequential*) and “Proportional to inspiratory and expiratory ventilator flow” (*Proportional*). Median accuracy of iNO concentration measured in the test lung was 2% (interquartile range, IQR -19; 36), -23% (IQR -29; -17) and 0% (IQR -2; 0) with *Continuous, I-Sequential and Proportional devices*, respectively. Increased ventilator flow-by resulted in decreased iNO concentration in the test-lung with *Continuous* and *I-Sequential* devices, but not with *Proportional* ones. NO_2_ formation measured to assess potential risks of toxicity never exceeded the predefined safety target of 0.5 ppm. However, NO_2_ concentrations higher than or equal to 0.3 ppm, a concentration that can cause bronchoconstriction, were observed in 19% of the different configurations.

**Conclusion:**

We identified three different generations of iNO-devices, based on their gas administration modalities, that were associated with highly variable iNO concentrations’ accuracy. Ventilator’s flow by significantly impacted iNO concentration. Only the *Proportional* devices permitted to accurately deliver iNO whatever the conditions and the ventilators tested.

**Supplementary Information:**

The online version contains supplementary material available at 10.1186/s13613-024-01351-w.

## Background

Nitric oxide (NO) is a free radical with a low molecular weight and a high affinity for oxygen (O_2_), whose strong vasodilator properties were described in the 80's [1, 2]. Because of a very short biologic half-life, inhaled nitric oxide (iNO) can be restricted to the pulmonary circulation preferentially in well-ventilated lungs areas. Thanks to this specific delivery administration, iNO may permit to improve gas exchange in case of ventilation/perfusion mismatch and/or limit right ventricular afterload in case of pulmonary hypertension without deleterious systemic vasodilation effect [3].

In adult intensive care units (ICU), inhaled vasodilators like NO are provided in roughly 8% of patients with acute respiratory distress syndrome (ARDS) and 13% when ARDS is severe [4], despite the lack of strong evidence-based guidelines [5–9]. A recent retrospective analysis of a large series of patients with COVID-19 associated ARDS reported an increased use of iNO (almost 20% of the patients) [10] with a possible benefit in the most severe patients. iNO improves oxygenation in 50% to 70% of mechanically ventilated patients [5, 11–18].

Several iNO-devices have been successively developed to administrate iNO, since the 80’s. The delivery could take place directly into the ventilator air inlet or into the inspiratory limb of the patient circuit [19–21]. New technologies developed by manufacturers were essentially driven by the need to obtain a stable and accurate concentration of iNO inside the inspiratory circuit [21–24] and to limit the risk of toxicity. Regarding the risk, both the formation of nitrogen dioxide (NO_2_) by contact of O_2_ with NO and the formation of methaemoglobin are potentially deleterious [25].

In this experimental in vitro study, after classifying the different iNO-devices commonly used nowadays based on their different working principles, we assessed their accuracy and potential risk of toxicity.

We hypothesized that the technical differences in iNO-devices and ventilator characteristics (as the flow-by that is highly variable from one ICU ventilator to another) could substantially affect the accuracy of iNO administration.

## Methods

### Lung Model

#### Bench settings

**Fig. 1:**
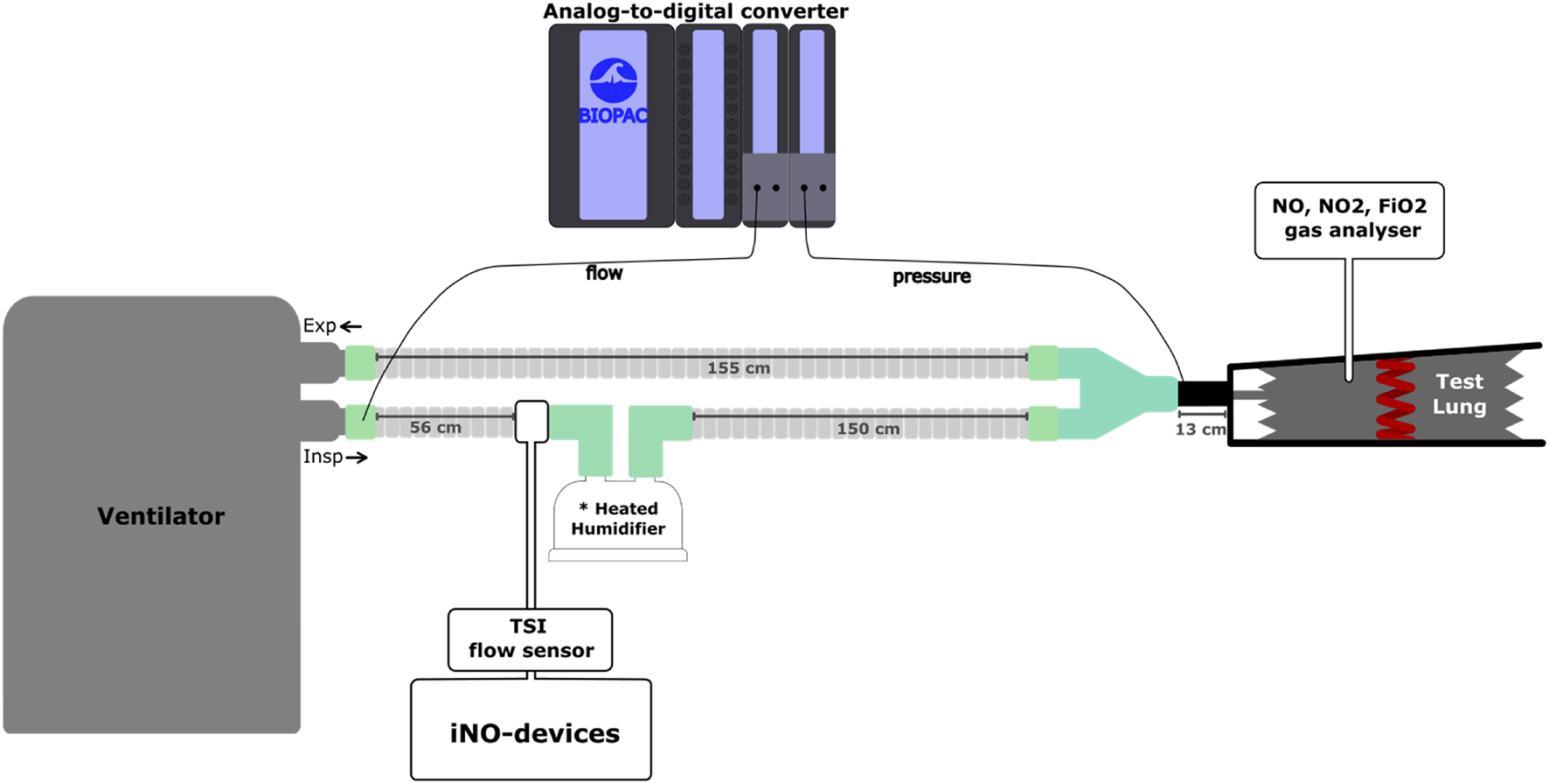
Description of the experimental bench model. The figure presents the bench set-up. The ventilators were connected to a Michigan test lung simulating mechanical ventilation in an ARDS patient. The patient circuit included a heated humidifier and the administration line of the iNO-device was connected to the humidifier inlet. The heated humidifier (MR290, Fisher and Paykel, Auckland, New-Zealand), with a volume of 280 mL, was full and at ambient temperature. A precise flow sensor was inserted on the administration line to record the NO flow delivered by each device. A pressure transducer was used to measure the airway pressure at the Y piece. A pneumotachometer was used to record the flow through the inspiratory limb of the patient circuit. The same specific breathing circuit, for heated humidifier, was used for each iNO-device and each ventilator (RT380, Fisher and Paykel, Auckland, New-Zealand). A majority of experiments has been done in the Vent’Lab (Angers university hospital) while some of them have been performed in the medical intensive care unit of the university hospital of Amiens

A Michigan test-lung (Michigan Dual Adult Test Lung, Michigan Instruments, Grand Rapids, Michigan, USA) was used to reproduce mechanical ventilation in ARDS condition, with a respiratory system compliance of 30 mL/cmH_2_O and resistances of 20cmH_2_O/L/s. The experimental bench setting is described in Fig. 1. The ventilator tested was connected to the test-lung via a double limb circuit with a heated humidifier. iNO-devices were implemented to provide NO in the inspiratory limb of the patient circuit.

#### iNO-devices and ventilators tested

We tested seven iNO-devices, the characteristics of which are shown in Table 1.

**Table 1:** Characteristics of iNO-devices tested

	Manufacturer	Pressure or flow sensor or cable	Electrochemical NO and NO_2_ analyzer available	Response time of the analyzer
INOmax DSIR	Mallinckrodt Pharmaceuticals, Dublin, Ireland	Flow sensor	Yes	30 s
Just Press	ITC, Madrid, Spain	NA	No	NA
MiniKINOX	Cahouet, Montreuil, France	NA	No	NA
NO-A	EKU Elektronik GmbH, Leiningen, Germany	Cable	Yes	10 s
NOX-tec	ITC, Madrid, Spain	Flow sensor	Yes	10 s
OptiKINOX	ALMS, Antony, France	Pressure sensor	No	NA
SoKINOX	iNOsystems, Antony, France	Flow sensor	Yes	10 s

*NA* Not applicable

Those iNO-devices were assessed with different ventilators offering different flow-by: Servo 900C (Siemens-Elema AB, Solna, Sweden) and Evita 4 (Dräger, Lübeck, Germany) without flow-by, SERVO-i (Getinge, Göteborg, Sweden) and Evita Infinity V500 (Dräger, Lübeck, Germany) with a flow-by of 2L/min, Engström Carestation and Carescape R860 (GE Healthcare, Madison, USA) with a flow-by of 10L/min, Elisa 500 (Löwenstein, Steinbach, Germany) with flow-by of 10 and 30L/min. The flow-by is a specific flow not participating in patient minute ventilation, through the patient circuit during the expiration phase, necessary to generate triggering and to improve non-invasive ventilation performances. Values of flow-by for various ventilators are detailed in the Additional data 1.

#### Data recording and analysis

As illustrated in Fig. 1, a pneumotachometer (3700 Series, Hans Rudolph Inc., Shawnee, USA) recorded the flow in the inspiratory limb of the patient circuit. A pressure transducer was connected at the Y-piece to measure the airway pressure. The NO/N_2_ flow administrated was measured directly from the device with a high accuracy flowmeter (Mass flowmeter 4140, TSI Incorporated, Shorview, USA).

All the signals (ventilator’s flow, airway pressure, NO/N_2_ flow) were converted to digital signals using an analog-to-digital converter (MP 150, Biopac systems Inc., Goleta, California, USA) and analyzed by a dedicated software (Acqknowledge version 5, Biopac systems Inc.) (Fig. 1).

NO and NO_2_ concentrations were measured directly in the test-lung, by the electrochemical analyzer of the SoKINOX (the analyzer has a response time of 10 s and the screen has a display refresh of 7.8 Hz). Supplementary tests have been performed using a chemiluminescent sensor (with a response time of 1 s) to validate the measurements performed using the electrochemical technique (see Additional data 2).

### Protocol

The ventilators were set in volume mode, with a tidal volume of 450 mL (corresponding to 6 mL/kg predicted body weight for an adult male, 180 cm tall), constant inspiratory flow of 60L/min, respiratory rate of 25 cycles/min, plateau time of 200 ms, positive end-expiratory pressure of 10cmH_2_O (to mimic end-expiratory lung volume), trigger sensitivity was set at the default value (so there was no auto-triggering). FiO_2_ was set at 100% to maximize the risk of NO_2_ formation.

Four iNO concentrations were tested: 5, 10, 14 and 20 ppm for each couple of iNO-device/ventilator. The settings for each iNO-device were performed based on the recommendations of the user manual with some additional tests when required (see Additional data 3 for details). The iNO administration site was similar whatever the iNO-device, see Fig. 1. When several working modes were available, the most advanced mode was chosen. However, the hot-wire flow sensor was not used for the NO-A.

All tests were performed at two initial cylinder concentrations of NO/N_2_ gas mixture: 450 and 800 ppm.

### Statistical analysis

The relative iNO concentration error (ΔNO) was defined as the relative difference between the effective iNO concentration measured in the test lung and the targeted iNO concentration in percentage. ΔNO was expressed as median and interquartile ranges (IQR) over four tested iNO concentrations (5, 10, 14 and 20 ppm) and two tested cylinder concentrations (450 and 800 ppm) according to the data availability expressed in the Additional data 4.

Performances of iNO-devices were considered as acceptable when ΔNO was within ± 20%, as indicated by the FDA guidance [26].

## Results

### Description of three different generations of iNO-devices

**Fig. 2:**
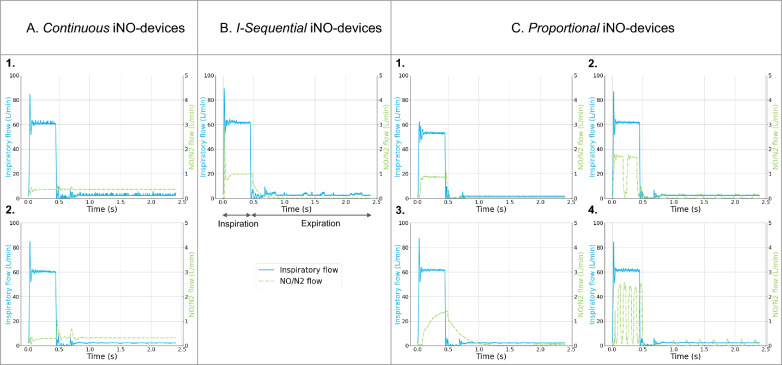
Classification of iNO-devices. Three generations of iNO-devices are described. For each type, one respiratory cycle is illustrated including inspiration and expiration. The blue curve is the inspiratory flow recorded through the inspiratory limb of the patient circuit. The dotted green curve is the NO/N_2_ flow recorded through the administration line. From the left to the right: **A** *Continuous* delivery: constant NO/N_2_ flow during both inspiratory and expiratory phases, the A.1 is Just Press and A.2 is MiniKINOX; **B** *I-Sequential* delivery: the NO/N_2_ flow is null during expiratory phase, the panel is OptiKINOX; **C** *Proportional* delivery: the NO/N_2_ flow increases during inspiratory phase and decreases but stays positive during expiratory phase, the C.1 is INOmax, C.2 is NO-A, C.3 is NOXtec and C.4 is SoKINOX

Based on the different patterns of iNO delivery among the seven devices tested, we identified three generations of iNO-devices (Fig. 2).

A. *Continuous* iNO-devices (Just Press, MiniKINOX): based on a simple flowmeter, this technology provides a constant and continuous NO/N_2_ flow during both inspiratory and expiratory. The NO/N_2_ flow is manually set on the flowmeter based on predefined tables taking into account the targeted iNO concentration, the patient minute-ventilation, the cylinder concentration and possibly the inspiratory time (Ti).

B. *Sequential to inspiration* or *I-Sequential* iNO-devices (OptiKINOX): this technology allows to administer a NO/N_2_ flow only during the inspiratory phase, whereas the NO administration is stopped during the expiratory phase. The NO flow is synchronized with the inspiratory phase by the detection of pressure variations inside the inspiratory limb of the patient circuit. The targeted iNO concentration, patient minute ventilation and Ti are set on the device by the clinician.

C. *Proportional to inspiratory and expiratory ventilator flow* or *Proportional* iNO-devices (INOmax, NO-A, NOXtec and SoKINOX): this technology allows to administer a NO/N_2_ flow proportional to ventilator flow going through the inspiratory limb of the patient circuit during both the inspiratory and expiratory phases. As a result, iNO delivery to the patient circuit differs during the inspiratory and expiratory phases. This adaptation of NO flow necessitates a continuous measurement or estimation of the minute ventilation either by a direct measurement on the inspiratory limb of the patient circuit or by electronic interface with the ventilator.

### Accuracy of iNO delivery according to the different iNO-device generations

**Fig. 3:**
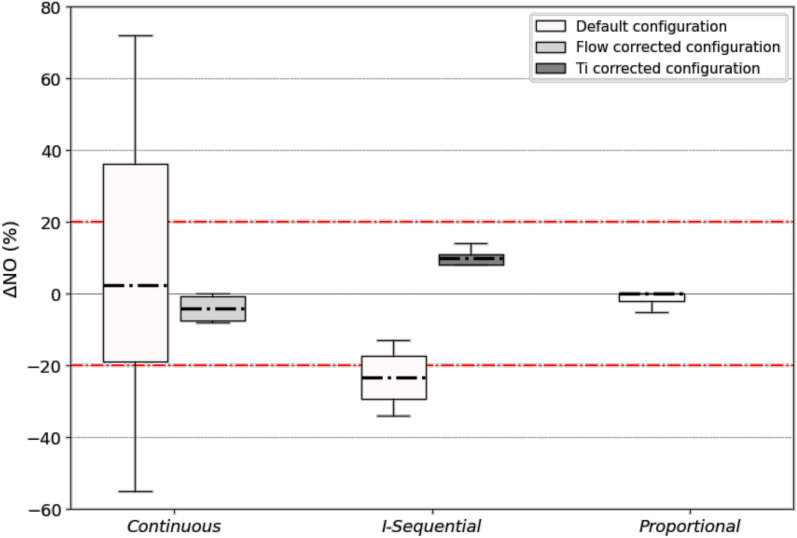
Relative iNO concentration error (ΔNO) with the three generations of iNO-devices. Box-plots represent median, interquartile, maximal and minimal values of ΔNO, for each generation of iNO-devices. The dotted red line represents the acceptable target range of iNO concentration error in relative percentage. Each box-plot includes four targeted iNO concentrations (5, 10, 14, 20 ppm) and two NO/N_2_ cylinder concentrations (450 and 800 ppm) when available. For default configuration, n = 16 for *continuous* devices, n = 12 for *I-Sequential* devices, and n = 32 for *proportional* devices. *Flow corrected configuration means adjustment of NO/N_2_ flow by a precise flow sensor to the calculated value of flow for *continuous* devices. *Ti corrected configuration means adjustment of Ti/Ttot ratio to the insufflation time for *I-Sequential *devices

The accuracy of iNO delivery for the three different generations of iNO-devices described above with a ventilator providing a flow-by of 2L/min is displayed in Fig. 3 and detailed in the additional data 5 by targeted concentrations.

ΔNO for *continuous* devices was low but a high dispersion was recorded (2%, IQR -19; 36%, n = 20). Actual iNO concentrations’ accuracies were significantly improved when the NO/N_2_ flow was adjusted using an external flow sensor that was more precise than the pointer of the iNO-devices (flow corrected configuration, Fig. 3). The adjusting method of the *continuous* iNO-devices can also impact delivery accuracy, as detailed in Additional data 6 and 7.

*I-Sequential* iNO-devices systematically resulted in an under-administration of iNO (ΔNO -23%, IQR -29; -17%, n = 12). The accuracy was improved when plateau time was excluded from the Ti set on the device (Ti corrected configuration, Fig. 3), as explained in the Additional data 8.

With *proportional* iNO-devices, ΔNO was systematically in the ± 20% error range (0%, IQR -2; 0%, n = 32).

NO_2_ formation never exceeded the predefined safety target of 0.5 ppm whatever the device and condition tested. Of note, we measured NO_2_ concentrations higher than or equal to 0.3 ppm in 19% of our configurations, which could cause bronchoconstriction [27].

The cylinder concentration had no significant impact on ΔNO (data not shown).

### Impact of the flow-by on iNO delivery

**Fig. 4:**
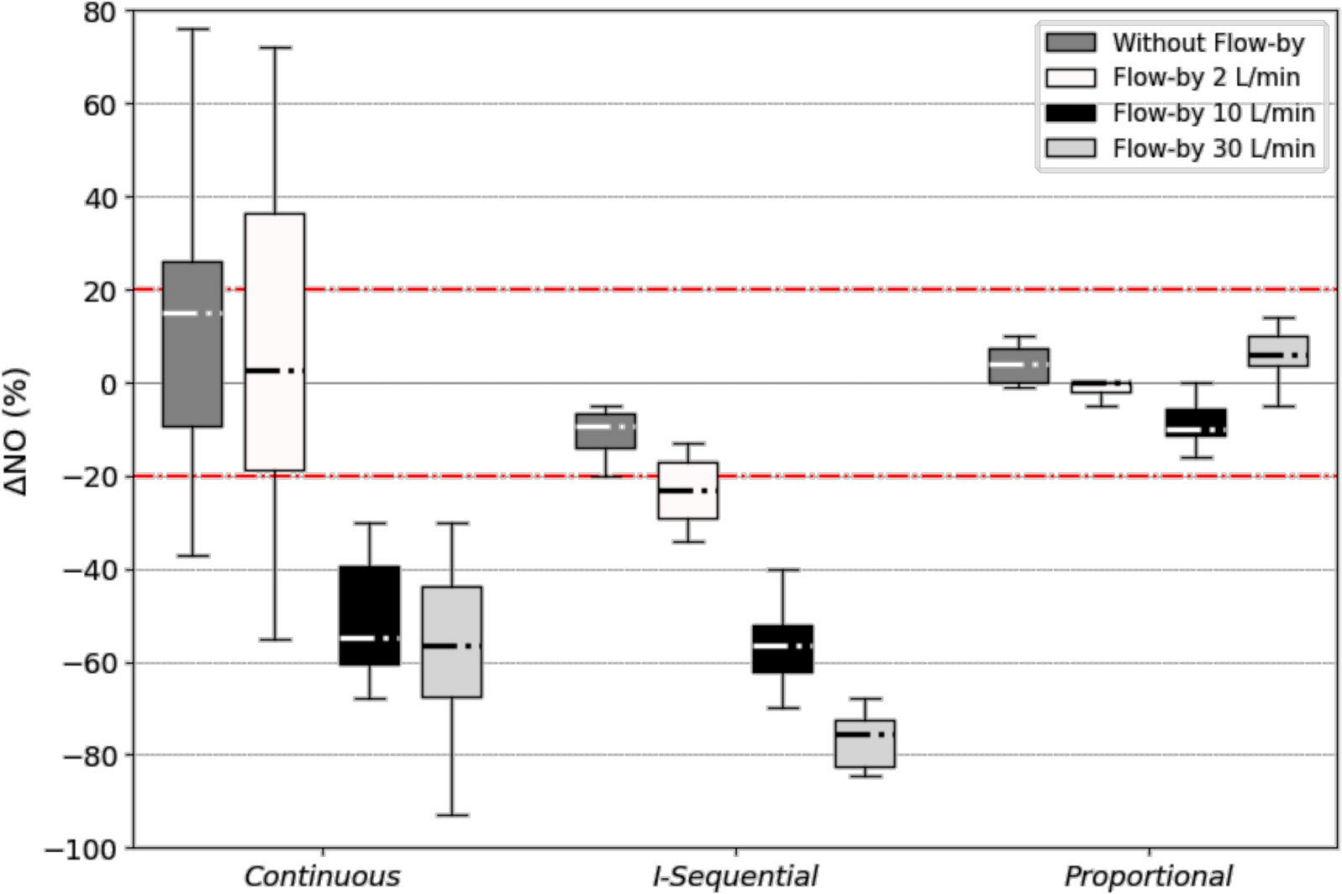
Impact of flow-by on relative iNO concentration error (ΔNO) in the three generations of iNO-devices. Box plots represent median, interquartile range, maximal and minimal values of ΔNO, for each generation of iNO-devices. Four flow-by values (0, 2, 10 and 30 L/min) were tested for each generation. The dotted red line represents the acceptable target range of iNO concentration error in relative percentage. Each box-plot includes four targeted iNO concentrations (5, 10, 14, 20 ppm) and two NO/N_2_ cylinder concentrations (450 and 800 ppm) when available. For *continuous* devices, n = 8 without flow-by, n = 16 for flow-by of 2 L/min, n = 16 for flow-by of 10 L/min and n = 12 for flow-by of 30 L/min. For *I-Sequential* devices, n = 4 without flow-by, n = 12 for flow-by of 2 and 10 L/min and n = 8 for flow-by of 30 L/min. For *Proportional* devices, n = 8 without flow-by, n = 32 for flow-by of 2 L/min, n = 20 for flow-by of 10 L/min and n = 12 for flow-by of 30 L/min

Among the four flow-by tested (0, 2, 10 and 30L/min), higher was the flow-by, higher was the under-delivery of iNO, except for the *proportional* iNO-devices (Fig. 4). *Proportional* devices were the only ones able to maintain iNO concentration within the target when flow-by increased.

Of note, the impact of iNO administration site on iNO delivery has been assessed in Additional data 9.

## Discussion

Present experimental in-vitro findings could be summarized as follows:

1. We proposed herein to classify commercially available iNO-devices in three technological generations (*Continuous, I-Sequential, Proportional*).
2. Their performances to deliver the targeted iNO concentrations in the test-lung were highly variable, depending on their working principle.
3. The adjunction of a flow-by (highly variable and often blind on ICU ventilators) resulted in a systematic and significant under-delivery of iNO with *Continuous* and *I-Sequential* generations of iNO-devices.
4. Only the *Proportional* devices allowed to maintain the NO concentration in the test lung within the predefined ± 20% error margins for all conditions tested including different ICU ventilators flow-by.

### Classification of iNO-devices

To our knowledge, a classification including the latest commercially available iNO-devices and taking into account the interactions with different ventilators has never been proposed. The classification that we propose herein describes and differentiates three different iNO administration patterns that variably affect iNO delivery accuracy. In addition, this classification is valuable for understanding the substantial impact of ventilators flow-by on iNO delivery. This concern has never been systematically investigated but deserves to be addressed because of the increasing variety and heterogeneity of ICU ventilators [28].

### Heterogeneity of accuracy performances among the different generations of iNO-devices

#### Continuous administration iNO-devices

iNO actually delivered and resulted iNO concentration reached with *continuous* devices were highly variable. This heterogeneity might be explained by the type of table and by the precision of the flowmeter used to adjust the NO/N_2_ flow.

Two types of tables are currently used to determine the NO/N_2_ flow necessary to reach the targeted iNO concentration (see Additional data 3). The “dilution method” takes into account the minute ventilation to estimate the dilution of NO/N_2_ flow. Differently, the “Ti method” integrates the Ti, considering that the gas delivered during the expiratory phase will not reach the lungs. The calculated NO flow is systematically higher with the “Ti method” than with the “dilution method” because iNO has to be administered over a shorter period of time; in return, a higher dose of iNO is also delivered during the expiratory phase in the inspiratory limb of the patient circuit. This results in significantly higher iNO concentrations and higher NO_2_ formation.

The insufficient precision of the flowmeter may also explain the heterogeneity of the results observed with continuous administration as shown by the improved iNO delivery performances when using an additional more sensitive flow sensor.

#### I-Sequential administration iNO-devices

*I-Sequential* devices resulted in a systematic under-administration of iNO. This observation is mainly explained by the integration in the iNO-device of the Ti (ventilator setting including the insufflation and the end-inspiratory occlusion times) instead of the insufflation time only, to estimate the NO-flow to deliver. *I-Sequential* devices deliver NO-flow whose value is calculated assuming a Ti (including the end-inspiratory occlusion time) equal to the insufflation time while the NO flow is stopped during the occlusion, leading to an under-administration of iNO, as illustrated in Fig. 3 (see Ti-corrected configuration) and Additional data 8. To manage this limitation, it is possible to set on the device the insufflation time instead of the Ti. This apparently unimportant detail complicates the management of severe patients in routine practice since an end-inspiratory occlusion permitting to continuously monitor plateau pressure is often used.

Additionally, with *Continuous* and *I-Sequential* systems, any direct or indirect change in the minute ventilation (due to changes in settings by the clinician or to patient spontaneous triggering) may impact the accuracy of iNO delivery.

#### Proportional administration iNO-devices

Based on our results, *proportional* devices appear to be the most precise systems. This observation is perfectly expected in the light of their specific working principle that continuously adapt the NO/N_2_ flow delivered in the patient circuit as a proportion of the flow actually delivered by the ventilator. This generation automatically adapts iNO delivery to any change in ventilator patterns, (either controlled or assisted ventilation) thus maintaining constant the iNO concentration in the ventilator circuit during both inspiratory and expiratory phases.

Noticeably, this last generation of iNO-devices required a complex calibration process before starting the treatment that may complicate their use in emergency situations.

### Impact of ventilator flow-by on iNO delivery

In most recent ICU ventilators, a flow-by is present, often variable, but usually blind and misunderstood by the clinicians (see Additional data 1). Our results demonstrate that the additional flow related to the flow-by directly affects the NO delivered close to the humidifier resulting in a systematic and significant washout of NO present in the inspiratory limb during the expiratory phase. This washout is directly proportional to the level of flow-by. As a result, NO concentration reached in the test-lung significantly decreased when the flow-by increased except with the last generation devices that measure the flow-by and automatically compensate it to maintain optimal iNO concentrations.

Interestingly, positioning iNO administration close to the Y-piece, on the inspiratory limb, prevents flow-by washout effect but is in turn associated with a systematic iNO under-delivery (see Additional data 9). This site of administration available only with *continuous* and *I-Sequential* iNO-devices permits to theoretically reduce the risk of NO_2_ formation This configuration requires higher NO flows to obtain the target concentration depending on the inspiratory flow set on the ventilator.

### Bolus effect

A phenomenon called “bolus effect” has been well described with *continuous* iNO-devices [23, 24]. It is defined as an over-administration of NO due to NO accumulation in the circuit during the expiratory phase and delivered to the patient at the beginning of the next inspiration. This accumulation may be more pronounced for *continuous* iNO-devices, as they continue to deliver NO flow in the circuit during the expiratory phase, which is not the case for *I-Sequential* iNO-devices. Interestingly, ventilators tested in these studies did not have flow-by. The substantial wash-out induced by the flow-by may explain, at least in part, that this bolus effect was not observed in our study. It should also be noted that the bolus effect may differ depending on the site of iNO administration. Similarly, the addition of a mixing chamber on the inspiratory limb of the circuit (such as a heated humidifier) may also have reduced this phenomenon [29].

### Limitations of the bench model

Our model has some limitations. First, it is an experimental inert model, without the ability to simulate NO uptake. This model has the benefit to be reproducible, whatever the iNO-devices tested and without temporal bias. Other models have been proposed to simulate NO uptake but present other limitations [24, 28]. As the tests were carried out with a heated humidifier in favor of iNO mixing, these results may not be generalized to a setting with a heat and moisture exchanger filter. iNO dosages in the present study were limited from 5 to 20 ppm since it represents the most commonly used dosages to treat ARDS patients. Different results could be observed when setting higher dosages and when using different ventilators in neonatology or during anesthesia. We chose to monitor NO and NO_2_ concentrations inside the test-lung to attempt to approach the most accurate concentrations we could observe inside a lung, but the experimental design of the study may have affected these measurements. Additionally, the technology used to monitor iNO in the present study was electrochemical analysis while chemiluminescence characterized by a higher sampling rate has also been widely used for this purpose. Importantly, additional tests confirmed the consistency of the NO concentration measured with chemiluminescence and electrochemical techniques. Finally, pressure regulated modes have not been specifically tested.

## Conclusion

Based on the present bench experiment, we describe three different generations of iNO-devices that exhibit heterogeneous results regarding iNO concentrations accuracy. The presence of flow-by is probably one of the most common and unrecognized causes of misadministration. Last generation of *proportional* devices are the only ones able to accurately deliver iNO whatever the conditions and ventilator settings tested.

## Ethics approval and consent to participate

Not applicable.

## Consent for publications

Not applicable.

## Competing interests

ML is PhD student attached to the Mitovasc Lab and the Vent'Lab (Angers University Hospital) and co-financed by Air Liquide Medical Systems. AV, CB, YZ, JMF and MLT declare no competing interest. AL is employed by Air Liquide Medical Systems. JCR received a part-time salary from Air Liquide Medical Systems as scientific director of the MedLab. NP is employed by iNOsystems. MS is employed by Air Liquide Santé International. AM received consulting fees from Air Liquide Medical Systems. FB reports personal fees from Löwenstein and Air Liquide Medical Systems and research support from GE healthcare, outside this work.

### Supplementary Information

Additional file

## Data Availability

The datasets used and analyzed during the current study are available from the corresponding author on reasonable request.

## Abbreviations

NO: Nitric oxide
ICU: Intensive care units
iNO: Inhaled nitric oxide
O_2_: Dioxygen
NO/N_2_: Nitric oxide in nitrogen gas mixture
NO_2_: Nitrogen dioxide
I-Sequential: Sequential to inspiratory phase
Proportional: Proportional to inspiratory and expiratory ventilator flow
IQR: Interquartile range
ARDS: Acute respiratory distress syndrome
FiO_2_: Inspired fraction of oxygen
ppm: Parts per million
FDA: Food and Drug Administration
Ti: Inspiratory time
