# Supplementary material for: Modes of administration of nitric oxide devices and ventilators flow-by impact the delivery of pre-determined concentrations
Source: Ann Intensive Care. 2024 Aug 21;14:130. doi: 10.1186/s13613-024-01351-w (PMC11339004; doi:10.1186/s13613-024-01351-w)
Supplement: Supplementary file 1 — Additional file [file 13613_2024_1351_MOESM1_ESM.docx]

**Additional data 1: Ventilator flow-by according to manufacturer’s informations**

| **Ventilator** | **Flow-by (L.min^-1^)** |
| --- | --- |
| Servo 900C (Siemens-Elema AB, Solna, Sweden) | 0 |
| Evita 4 / Evita XL (Dräger, Lübeck, Germany) | 0 |
| Servo-I / Servo-U (Getinge, Göteborg, Sweden) | 2 |
| Infinity V500 / V800 (Dräger, Lübeck, Germany) | 2 |
| Avea (CareFusion, Yorba Linda, CA, USA) | Adjustable :  0.4 - 5 |
| C3 / C6 (Hamilton, Bonaduz, Switzerland) | 6 |
| Bellavista 1000 / 1000e (imtmedical, Buchs, Switzerland) | 6 |
| Engström Carestation / Carescape R860  (GE Healthcare, Madison, USA) | Adjustable : 2.5 - 10 |
| SV300 / SV600 / SV800 (Mindray, Shenzhen, China) | Variable :  3 - 20 |
| Puritan Bennett 840 / 980 (Medtronic- Covidien, Mansfield, USA) | Variable:  1.7 - 21.5 |
| G5 / S1 (Hamilton, Bonaduz, Switzerland) | Variable:  1 - 30 |
| Elisa 600 / 800 (Löwenstein, Steinbach, Germany) | Adjustable : 3 - 30 |
| Elisa 300 / 500 (Löwenstein, Steinbach, Germany) | Adjustable : 10 - 30 |

**Additional data 2: Difference on NO concentrations measured by electrochemical or chemiluminescent analyser**

NO concentrations were recorded inside the test lung by electrochemical and chemiluminescent analyser (with a time response of 1s).


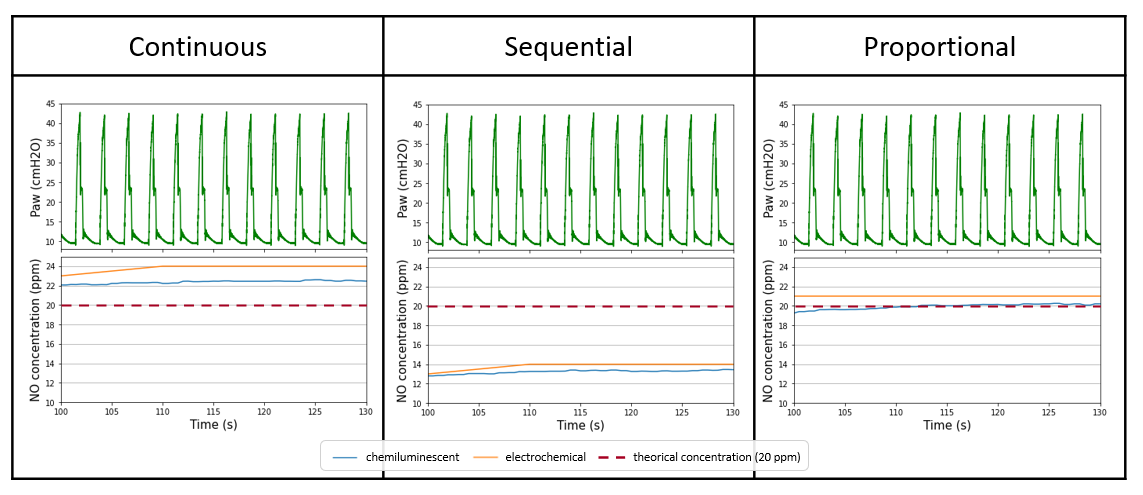


**NO concentrations inside the test lung, depending on the analyser and the iNO-device generation.**

For each generation devices, the airway pressure (Paw) is shown in green (upper panel) and the NO concentration measured by electrochemical analyser in orange and by chemiluminescence analyser in blue (lower panel). The target concentration is 20 ppm (red dotted line).

**Additional data 3: iNO-devices settings**

The continuous flowmeter (MiniKINOX) was set according the two different methods existing.

The first method, called “Dilution method” used the following formula:

1. $NO flow =\frac{[NO]patient * VM}{[NO]cylinder}$

- $NO flow$ is the flow set on the device.
- $\left[ NO \right]patient$ is the iNO targeted concentration.
- $VM$ is the minute ventilation.
- $[NO]cylinder$ is the cylinder concentration of NO/N_2_ gas. The usual cylinder concentrations are 450 ppm and 800 ppm.

The second method, called “Ti method” used the following formula [^[[1]](#footnote-1)^]:

1. $NO flow =\frac{[NO]patient * Ttot * VM}{[NO]cylinder * Ti}$

- $Ttot$ is the total respiratory time (= 60/respiratory rate).
- $Ti$ is the insufflation time, not including the plateau time.

Corresponding NO flows calculated with these two formulae, for a cylinder concentration of 450 ppm, were grouped in the following table:

| NO concentration (ppm) | NO flow calculated by “dilution method” (L.min^-1^) | NO flow calculated by “Ti method” (L.min^-1^) |
| --- | --- | --- |
| 5 | 0.12 | 0.67 |
| 10 | 0.25 | 1.33 |
| 14 | 0.35 | 1.87 |
| 20 | 0.5 | 2.66 |

To ensure the consistency of the results, we performed additional tests by adjusting the NO/N2 flow (according to the table above) using an external and more precise flow sensor.

The calibrated continuous flowmeter (Just Press) was set according to abacuses given by the constructor, according to the cylinder concentration of NO/N_2_ gas.


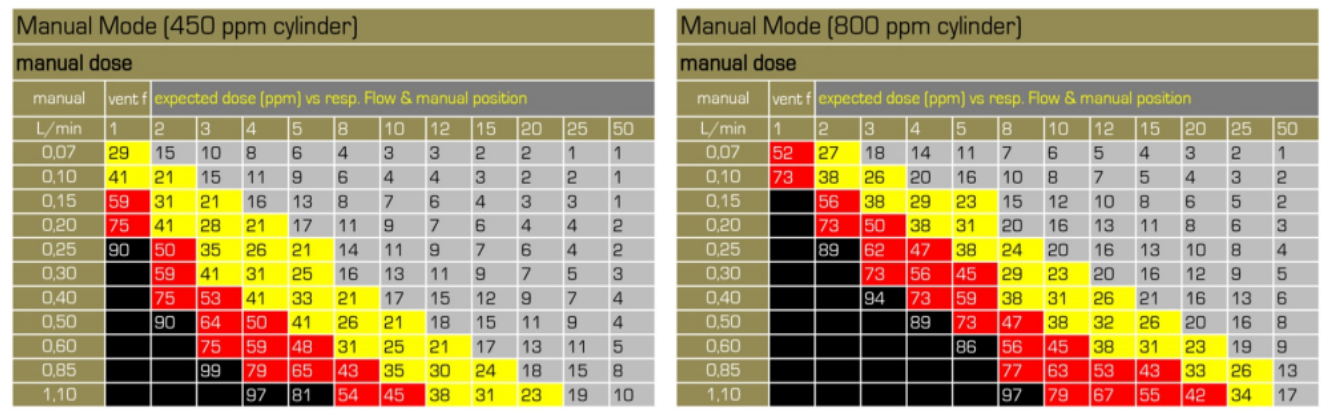


We consulted these abacuses on [Ingeniería y Técnicas Clínicas (itcsal.com)](http://eng.itcsal.com/downloads-section), Regulators and flowmeters, Ed. March 2021 (<http://www.eng.itcsal.com/downloads-section>).

These abacuses are based on the "Dilution method” formula.

The “I-Sequential” iNO-devices (OptiKINOX) is configured in synchronized mode. The operator set the targeted iNO concentration, the minute ventilation and the inspiratory/expiratory ratio (Ti/Ttot or I/E).

iNO concentration was set to 5, 10, 14 or 20 ppm. The minute ventilation was near the calculated patient minute ventilation: 11 L.min^-1^. Ti/Ttot ratio was set to 27%. To ensure the consistency of the results, we performed additional tests by adjusting the Ti/Ttot ratio to 19%, excluding the plateau time in the Ti.

Four “Proportional” iNO-devices were tested.

One device (NO-A) synchronizes its delivery through connection to the ventilator with a RS232 cable. Where possible, the operating mode was synchronized for the compatible ventilator. For others, despite the connection, the delivery could only be continuous, adapted to the total flow obtained from the ventilator by the RS232 cable. The targeted iNO concentrations were set to 5, 10, 14 and 20 ppm.

Three other devices (INOmax, NOXtec and SoKINOX) were used in real-time (or auto-sense) mode. Only the targeted iNO concentration was set.

**Additional data 4: Data availability**


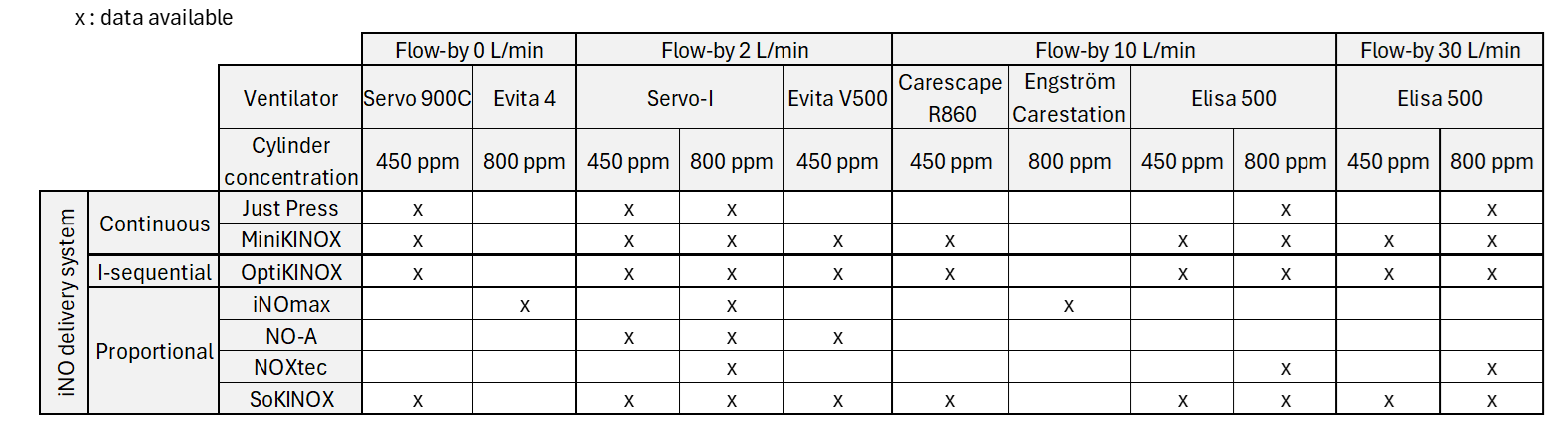


This table presents the data availability according to the ventilator, the cylinder concentration and the iNO-devices.

**Additional data 5: Accuracy of NO delivery for the different iNO-device generations and targeted NO concentration**


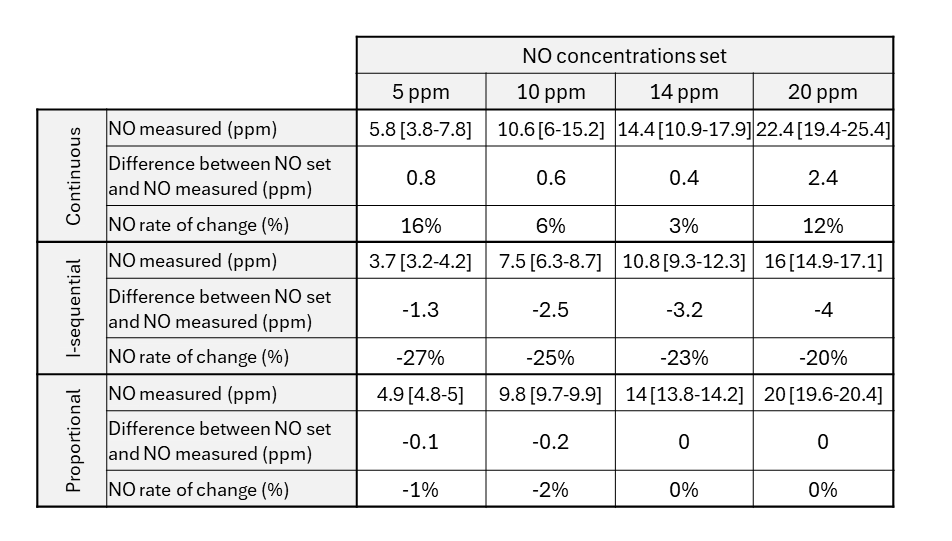


This table presents the accuracy of NO delivery for the different iNO-device generations according to each targeted NO concentration for each set of data available with flow-by of 2 L.min^-1^. NO concentrations are expressed as mean with 95% confidence interval.

**Additional data 6: Impact of the dilution formula for continuous devices**


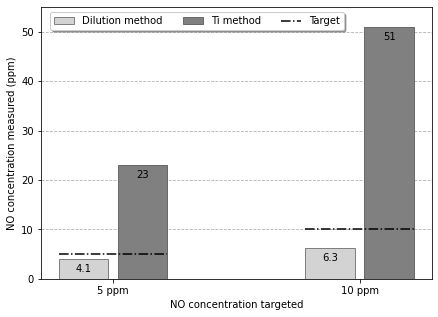


**NO concentration depending on the formula of dilution used for continuous iNO-devices.**

The figure represents the actual concentration of NO in the test-lung for the two formula of dilution used for the continuous devices: “Dilution method” and “Ti method”, for two target NO concentrations (5 and 10 ppm). The flow-by is 2 L.min^-1^.

**Additional data 7: Calibrated flow or measured flow**

| **Calibrated flow* (L.min^-1^)** | **Measured flow** (L.min^-1^)** | **Difference**  **(L.min^-1^)** |
| --- | --- | --- |
| 0.07 | 0.11 | 0.04 |
| 0.1 | 0.14 | 0.04 |
| 0.14 | 0.25 | 0.11 |
| 0.19 | 0.31 | 0.12 |
| 0.25 | 0.41 | 0.16 |
| 0.33 | 0.49 | 0.16 |
| 0.41 | 0.56 | 0.15 |
| 0.5 | 0.66 | 0.16 |
| 0.61 | *0.78 | 0.17 |
| 0.85 | 1 | 0.15 |
| 1.12 | 1.23 | 0.11 |

* Calibrated flow corresponds to the flow indicated by the calibrated orifices of the flowmeter.

** Measured flow is recorded by a high accuracy flowmeter (Mass flowmeter 4140, TSI Incorporated, Shorview, USA).

**Additional data 8: I-Sequential devices**


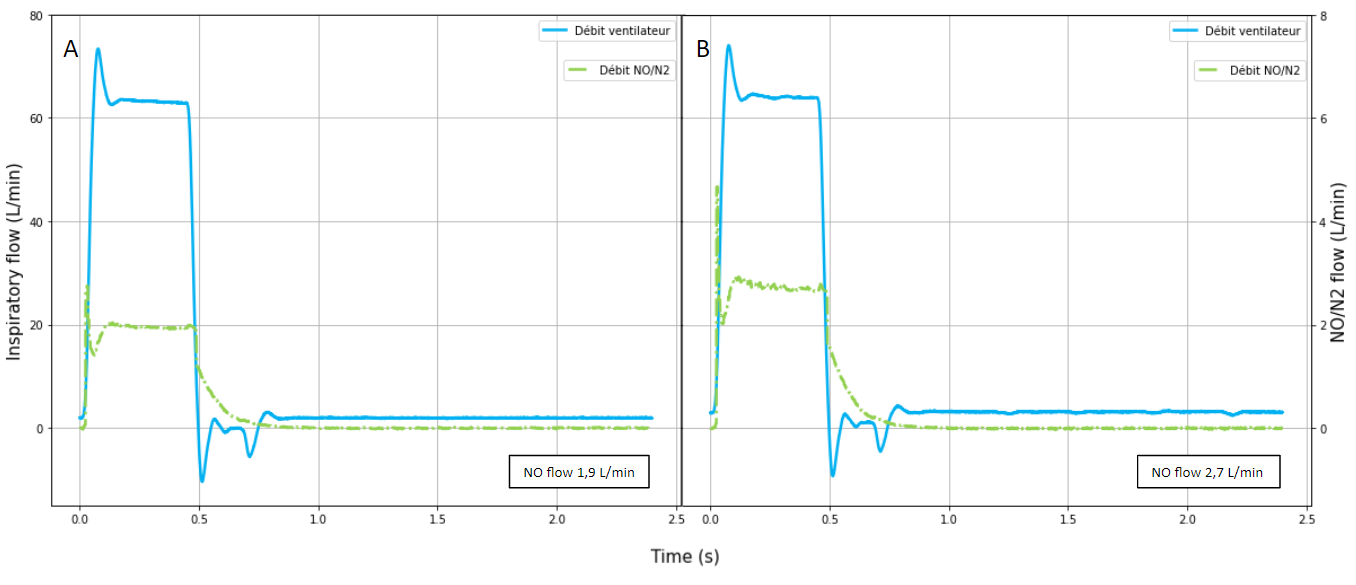


**Variations of NO/N2 delivery by I-Sequential devices, in response to flow**

The Ti/Ttot ratio set on the device is modified from 27% to 19%, the NO concentration set is not modified (20 ppm). The tidal volume, the inspiratory flow and the respiratory rate are unchanged (450 mL, 60 L.min^-1^ and 25 cycles.min^-1^).

The NO/N2 gas flow (green dotted curve) delivered by the device increases while the inspiratory time is unchanged. Change of Ti/Ttot ratio set on the iNO-devices modifies the delivered NO flow but not the delivery time.

**Additional data 9: Impact of the iNO administration site**


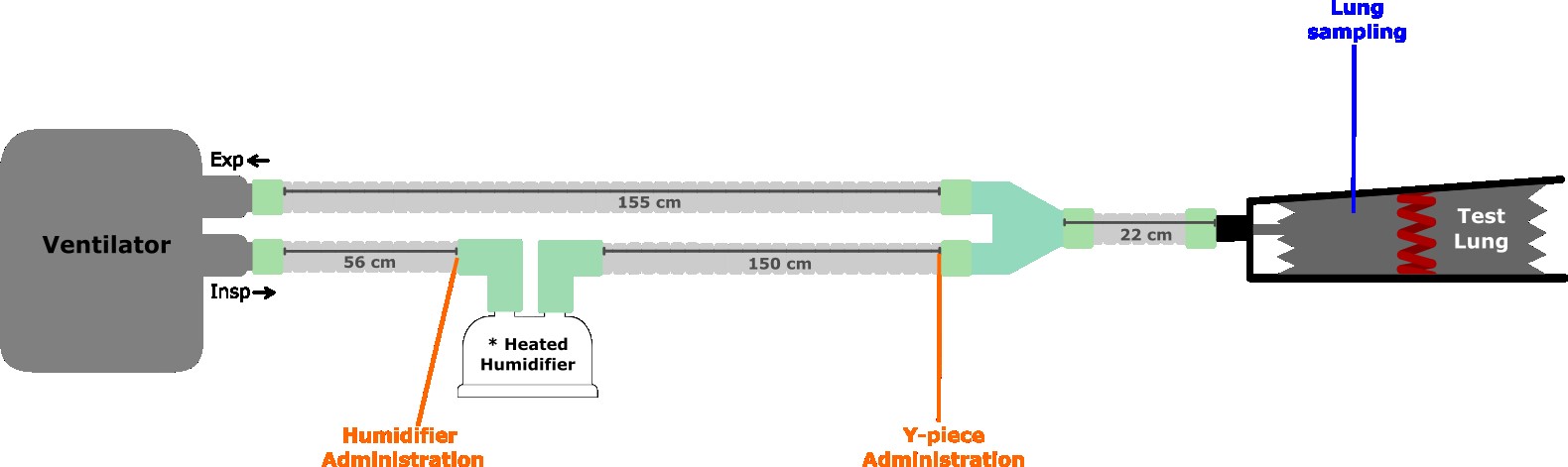


**Bench model and location of NO administration (two configurations tested)**

Two different NO administration locations were tested: close to the heated humidifier, close to the Y-piece in the inspiratory branch. NO concentrations were measured inside the test lung.


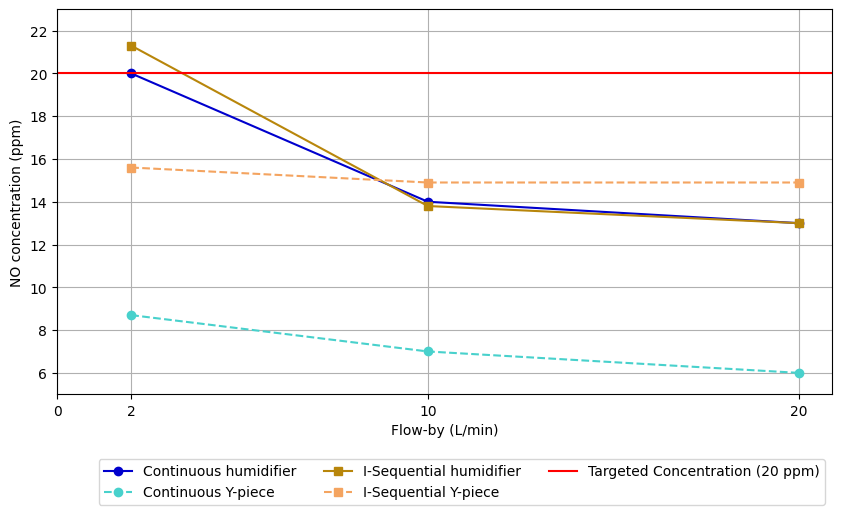


**NO concentrations measured inside the test lung in different administration sites and iNO-device generations for three ventilators’ flow-by (2, 10 and 20 L/min) (targeted iNO concentration = 20 ppm).**

Red curve represents the targeted iNO concentration (20 ppm).

The solid curves represent the iNO concentrations measured in the test lung with an iNO administration at the humidifier, in circle blue for Continuous mode and in square brown for I-Sequential.

The dotted curves represent the iNO concentrations measured in the test lung with an iNO administration close to the Y-piece on the inspiratory limb, in circle blue for Continuous mode and in square brown for I-Sequential.

1. . Wysocki M, Delclaux C, Roupie E, Langeron O, Liu N, Herman B, et al. Additive effect on gas exchange of inhaled nitric oxide and intravenous almitrine bismesylate in the adult respiratory distress syndrome. Intensive Care Med. 1994;20:254–9. [↑](#footnote-ref-1)
